# The Accumulated Response of Deciduous *Liquidambar formosana* Hance and Evergreen *Cyclobalanopsis glauca* Thunb. Seedlings to Simulated Nitrogen Additions

**DOI:** 10.3389/fpls.2019.01596

**Published:** 2019-12-20

**Authors:** Zhenzhen Zhang, Yamin Zhao, Xiaoyan Zhang, Sichen Tao, Xiong Fang, Xingwen Lin, Yonggang Chi, Lei Zhou, Chaofan Wu

**Affiliations:** ^1^College of Geography and Environmental Sciences, Zhejiang Normal University, Jinhua, China; ^2^Fujian Provincial Key Laboratory of Soil Environmental Health and Regulation, College of Resources and Environment, Fujian Agriculture and Forestry University, Fuzhou, China

**Keywords:** evergreen and deciduous species, nitrogen addition, biomass, hydraulic changes, gas exchange

## Abstract

Nitrogen depositions in the Yangtze River Delta have is thought to shift the coexistence of mixed evergreen and deciduous species. In this study, the seedlings of the dominant evergreen species *Cyclobalanopsis glauca* Thunb. and the deciduous species *Liquidambar formosana* Hance from the Yangtze River Delta were chosen to test their responses to simulated N additions using an ecophysiological approach. N was added to the tree canopy at rates of 0 (CK), 25 kg N ha^−1^ year^−1^ (N25), and 50 kg N ha^−1^ year^−1^ (N50). The leaf N content per mass (*N*_m_, by 44.03 and 49.46%) and total leaf chlorophyll content (*Chl*, by 72.15 and 63.63%) were enhanced for both species, and *C. glauca* but not *L. formosana* tended to allocate more N to *Chl* per leaf area (with a higher slope). The enhanced N availability and *Chl* promoted the apparent quantum yield (*AQY*) significantly by 15.38 and 43.90% for *L. formosana* and *C. glauca*, respectively. Hydraulically, the increase in sapwood density (*ρ*) for *L. formosana* was almost double that of *C. glauca*. Synchronous improved sapwood specific hydraulic conductivity (*K*_S_, by 37.5%) for *C. glauca* induced a significant reduction in stomatal conductance (*g*_s_) (*p* < 0.05) in the N50 treatments, which is in contrast to the weak varied *g*_s_ accompanied by a 59.49% increase in *K*_S_ for *L. formosana*. As a result, the elevated maximum photosynthesis (*A*_max_) of 12.19% for *L. formosana* in combination with the increase in the total leaf area (indicated by a 37.82% increase in the leaf area ratio-leaf area divided by total aboveground biomass) ultimately yielded a 34.34% enhancement of total biomass. In contrast, the *A*_max_ and total biomass were weakly promoted for *C. glauca*. The reason for these distinct responses may be attributed to the lower water potential at 50% of conductivity lost (*P*_50_) for *C. glauca*, which enables higher hydraulic safety at the cost of a weak increase in A_max_ due to the stomatal limitation in response to elevated N availability. Altogether, our results indicate that the deciduous *L. formosana* would be more susceptible to elevated N availability even if both species received similar N allocation.

## Introduction

Evergreen and deciduous broad-leaved tree species can coexist across a variety of landscapes around the globe and play important roles in forest structure and functions (Wang et al., 2007; Kikuzawa et al., 2013; Ouédraogo et al., 2016). In these coexistent ecosystems, they usually differ in ecological habits, which contributes to the explanation of the mechanisms by which these two groups coexist (Xie et al., 2012; Devi and Garkoti, 2013; Álvarez-Yépiz et al., 2017). In recent decades, the fertilization effect of nitrogen deposition in forest ecosystems has received increasing attention (Wang and Feng, 2005; Zhang, 2006) and has been recognized as a threat to plant diversity in these mixed forests (Bobbink et al., 2010; Hietz et al., 2011; Liu et al., 2013a; Lu et al., 2014). As reported, the global nitrogen deposition in the end of last century was already exceeded 25 kg^−1^ ha^−1^ yr^−1^ (Binkley et al., 2004), and will be doubled in the end of this decade (Deutsch and Weber, 2012). However, the underlying mechanisms have rarely been discussed in previous studies (Takashima et al., 2004; Palmroth et al., 2014). Evergreen and deciduous species usually specialize their habitats and survival strategies for the tradeoff between photosynthetic capacity and persistence (Takashima et al., 2004; Shipley et al., 2006; Curtis and Ackerly, 2008). This specialization could enable the deciduous species with higher demands for nitrogen acquisition to photosynthesize more efficiently and allow evergreen trees to invest more N in the durable leaves that can persist through disturbances (Pringle et al., 2011). The existing evidence across 231 evergreen species and 110 deciduous species has already shown higher N enrichment for the latter due to the added N (Xia and Wan, 2008). Thus, we could expect that the deciduous species would also accumulate more leaf N than the evergreen species in the coexistent ecosystems. In addition, the evergreen and deciduous species also differ in the fraction of N investment in photosynthesis. Evidence has indicated that the photosynthetic N use efficiency (PNUE) is much lower for evergreen species than for deciduous species, which was primarily ascribed to the smaller fraction of nitrogen allocated to the photosynthetic apparatus, such as chlorophyll content, in evergreen species (Takashima et al., 2004; Hu et al., 2008). Comparably higher nitrogen content and specific leaf area (SLA) for the deciduous species enable their stronger morphology and photosynthetic plasticity (Hu et al., 2008). Therefore, this evidence supports the increased opportunity for deciduous species to take advantage of high N conditions (Takashima et al., 2004).

However, uncertainties still exist because disturbances such as seasonal drought could negate the fertilization effect for deciduous species by removing the ability to utilize elevated N (Wang et al., 2012a; Liu et al., 2013b). The underlying physiological reasons may be attributed to the different hydraulic tolerances in response to drought (Pivovaroff et al., 2016). The deciduous species possessed hydraulic architecture typical of drought-sensitive plants, i.e., low wood density, wider xylem vessels, higher sapwood-specific hydraulic conductivity (*K*_S_), and high vulnerability to drought-induced embolism (Choat et al., 2004; Kröber et al., 2015). In contrast, the evergreen species had lower *K*_S_ and leaf specific conductivity but were less susceptible to embolism (Choat et al., 2004; Chen et al., 2008). These differences would lead to stronger stomatal limitations for the deciduous species in dry conditions (Brodribb et al., 2002; Zhang et al., 2013; Kröber and Bruelheide, 2014). In addition, due to the fertilization effect, elevated photosynthesis leads to fast growth as well as decreased wood density and increased stem hydraulic conductivity (Pivovaroff et al., 2016), which potentially enhances the vulnerability of deciduous species in response to seasonal drought (Bauer et al., 2001; Wang et al., 2012b).

As a developing country during past decades, China has also experienced the most severe atmospheric nitrogen deposition in the world due to the rise of anthropogenic nitrogen release (Liu et al., 2013b; Lu et al., 2018). In particular, the Yangtze River Delta region, where urbanization is highly developed, is crucial for nitrogen deposition (Liu et al., 2013b), since the nitrogen deposition rates reached an level of 38.4 ^kg ha−1 yr−1^ in the end of last century (Zhou et al., 2001). In this study, we focused on the morphological and physiological responses of deciduous and evergreen species to the abundance of nitrogen to investigate whether the former could take advantage of the fertilization effect more fully due to seasonal drought disturbance. We hypothesized that:

1. The resource acquisition and utilization strategies of deciduous species will make them more susceptible to the added N, thus leading to a higher increase in leaf N and chlorophyll content than observed in the evergreen species.
2. Meanwhile, the increased stem conductivity and decreased wood density of deciduous species will lead to stronger stomatal limitation, which will neutralize the increase in leaf photosynthesis as well as the overall biomass.

Understanding the effects of N deposition on plant physiological processes for deciduous and evergreen species may illuminate the mechanisms behind overall forest responses to global changes.

## Materials and Methods

### Site Descriptions

**Figure 1:**
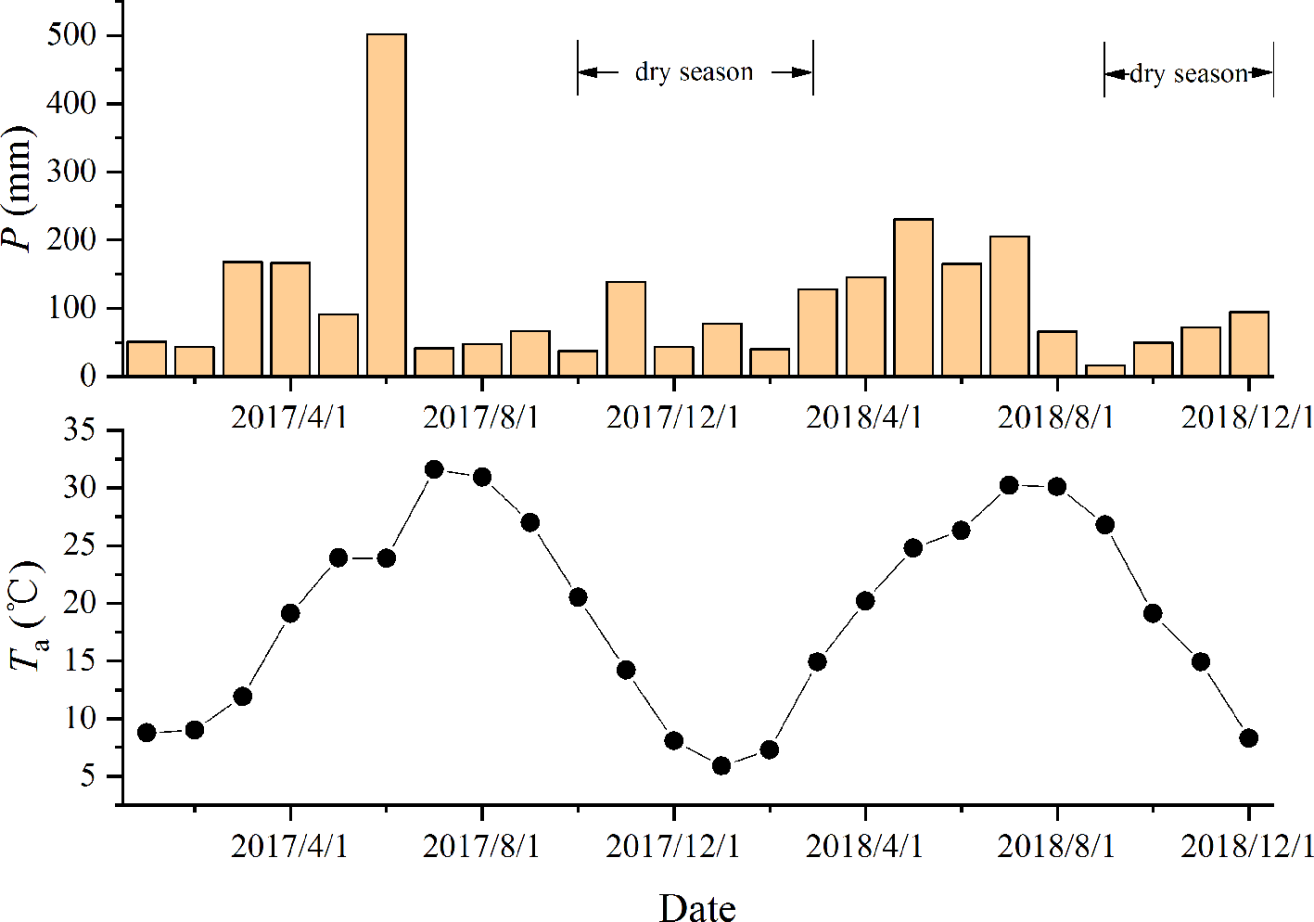
The dynamics of the monthly precipitation (*P*, mm) and mean temperature (*T*_a_) during the period (2017.1–2018.12). Data were obtained from the National Meteorological Information Center (http://data.cma.cn).

The study was conducted in the Dashu Garden in Jinhua, Zhejiang Province (119.79°E, 29.16°N), which is characterized by a subtropical monsoon climate with an average annual temperature of 17.3°C and an average annual precipitation of 1,300–1,600 mm. The elevation was 163 m and the soil was typical yellow soil. Located at the margin of temperate and subtropical zones, the vegetation is mostly covered by evergreen and deciduous broad-leaved mixed forests. The dry season starts in September and continues into March of the next year (**Figure 1**). In January 2017, seedlings of two dominant species, *Cyclobalanopsis glauca* Thunb. (evergreen broad-leaved) and *Liquidambar formosana* Hance (deciduous broad-leaved) were planted in holes (50 cm in diameter and 50 cm in depth) to carry out the simulated nitrogen addition experiments. In order to manipulate the original growth habitats of the two species more closely and ensure the water uptake from the deep soil, the typical mountainous red-yellow soil was moved from the secondary bare land in North Mountain, which was 1 km away from our site, and fully mixed into planting substrate in the hole.

Current N deposition rate in tropical forests of southern China is reported to range from 15 to 73 g N m^−2^ a^−1^ (Zhang et al., 2008), while the total deposition in our site was 2.69 g N m^−2^ a^−1^ (Xu et al., 2015). To simulate natural nitrogen deposition that might occur in the future, three treatments were designed, including the control group (CK), 25 gN m^−2^ a^−1^ (N25), and 50 gN m^−2^ a^−1^ (N50) (nitrogen from atmospheric deposition was not included); each had five replicates. Each replicate consisted of 10 individuals of 2-year-old seedlings. The experiment was started in April 2017. The NH_4_NO_3_ solutions were evenly sprayed from the canopy at the middle and the end of each month until October 2018. During the period of defoliation for *L. formosana* (November 2017 to March 2018), the NH_4_NO_3_ solution was sprayed onto the surface of the soil. In order to exclude the influence of changed water -regime, no extra water was input to the plots except for the rain. Weeding was carried out during the experiment to minimize the interference caused by other factors. After the N treatment was stopped, the leaves of both species were in good condition, indicating that the leaves were not significantly damaged.

### Gas Exchange and Leaf Water Potential

The gas exchange measurements were carried out from 9:30 to 11:30 on typical sunny days from October 11 to October 20, 2018. The photosynthetic light response curve was measured in the fully developed mature leaves (*n*=15 for each treatment) using a portable photosynthesis system (LiCOR 6800, LiCOR Biosciences, Lincoln, NE, USA). The light intensity intervals were set as 0, 100, 200, 400, 600, 800, 1,000, 1,200, 1,500 µmol·m^−2^·s^−1^, and the temperature was 25°C. The flow rate was set at 500 µmol s^−1^, and the CO_2_ concentration was set at 500 µmol·mol^−1^. We use a nonorthogonal hyperbolic model to obtain the light response curve parameters:

(1) $$A=\frac{1}{2\theta }\left(\text{AQYI}+A_{\text{max},g}-\sqrt{{\left(\text{AQYI}{+}_{\text{max},g}\right)}^{2}-4\theta \text{AQYI}A_{\text{max},g}}\right)-R_{d}$$

*A* is the CO_2_ gas exchange rate (µmol CO_2_ m^−2^s^−1^), *I* is the instantaneous light intensity (µmol m^−2^ s^−1^), apparent quantum yield (*AQY*) is the apparent quantum yield, *A*_max,g_ is the assimilation rate under saturating light (µmol CO_2_ m^−2^s^−1^), *θ* is the curvature, and *R*_d_ is the dark respiration rate (the CO_2_ exchange rate at *I*=0, µmol CO_2_ m^−2^s^−1^). We also recorded stomatal conductance (*g*_s,_ mol m^−2^ s^−1^) and intercellular CO_2_ concentrations (*C*_i_, µmol m^−2^ s^−1^).

Daily courses of leaf instantaneous net photosynthetic rate (*A*_i_, µmol CO_2_ m^−2^s^−1^)), *g*_s_, and transpiration rate (*E*_t_, µmol m^−2^ s^−1^) were measured at 2 h intervals from 5:00 am to 17:00 pm during October 22 to October 25 on 5 seedlings of each N treatment. Meanwhile, leaf water potential (*Ψ*_L_, Mpa) of the detached shoots was synchronously measured with a pressure chamber (PMS, Albany, OR, USA) after the instantaneous gas exchange measurements.

### Hydraulic Conductivity and Sap Wood Density

The current year branches were cut off (30 cm length) from each sampled tree (*n*=15). All branches were covered with black plastic bags to prevent water loss before they were immediately transported to the laboratory to determine the sapwood hydraulic conductivity (*K*_S_, g cm MPa^−1^ min^−1^ cm^−2^) with a high pressure flow meter (HPFM Gen3; Dynamx Corp., Elkhart, Indiana, USA). The specific experimental operations are as follows:

First, all the samples were cut off underwater at approximately 5 cm from the base. Then, the tree bark was removed approximately 3 cm from the sample ends. Emboli were removed from samples by vacuum infiltration under solution consisting of 0.22 µm filtered and degassed, distilled water for 8 h before being connected to HPFM under purified water (Barnard et al., 2011). Under the quasi-steady-state mode, deionized water purified by the Water Purification System (Milli-Q Advantage, Merk Millipore, Germany) was degassed and injected into the branches through HPFM at a pressure of 0.5 MPa until a stable flow rate appeared (approximately 5 to 10 min) to obtain the maximum whole-shoot level hydraulic conductance (*K*_h_, g cm MPa^−1^ min^−1^). After the measurements, the base diameter (*d*, mm) was measured with a vernier caliper to obtain the cross-sectional area (*A*_S_, cm^2^) of the base of the shoot, and the maximum sapwood hydraulic conductivity *K*_S_ (g cm MPa^−1^ min^−1^ cm^2^) was calculated as:

(2) $$K_{S}=K_{h}/A_{s}$$

Five segments of 3 cm length for each branch were collected to measure the sap wood density (ρ, g cm^−3^). The fresh volume (V, cm^3^) of these wood segments was determined gravimetrically by water displacement according to Archimedes’ principle after removing the bark and phloem (Osazuwa-Peters and Zanne, 2011). All samples were then oven-dried at 105°C for 24 h to get the dry weight (G, g). The sapwood density was calculated as ρ = G/V.

Stem xylem vulnerability was measured using the centrifuge method (Cochard et al., 2005; Sperry et al., 2012). After determining the maximum *K*_S_, a centrifugal machine (Sorvall RC-5C; Thermo Fisher Scientific, Waltham, MA, USA) equipped with a custom rotor (Alder et al., 1997) that was able to spin the stem segments was used to induce negative xylem pressure and induce cavitation. Hydraulic conductivity was measured between each induced pressure, and the percent loss of conductivity under a certain negative pressure (*PLC*_i_, %) was calculated as:

(3) $$\text{PL}C_{i}=100\times K_{\mathrm{hi}}/K_{\max}$$

where *K*_hi_ (g cm MPa^−1^ min^−1^) and *K* *_m_*_ax_ (g cm MPa^−1^ min^−1^) refer to the *K*_h_ at certain negative pressures and maximum *K*_h_. Vulnerability curves were constructed by plotting pressure *versus PLC*_i_ and fitting a Weibull model (Pammenter and Van der Willigen, 1998):

(4) PLC=100/(1+exp⁡(a(Ψ−b)))

According to this equation, water potential at 50% of conductivity lost (*P*_50_, MPa^−1^) was determined for each species.

### The Leaf Economic Traits

One hundred foliage round pieces were collected for 10–20 mature leaves with a hole puncher (10 mm in diameter) for each species in each treatment and were placed in the oven in an envelope for 24 h. The foliage rounds were weighed to estimate the (cm^2^ g^−1^). The remaining leaves in another envelope B. Five to 10 leaves were taken from the remaining leaves of each shoot and cut into slices with a width of 0.5 mm (0.2–0.5 g). The leaf chlorophyll of these samples was extracted with a mixed solution (acetone:ethanol = 2:1) for approximately 24 h until they turned white. Then, a spectrophotometer was used to measure the absorbance of the supernatant liquid. The calculation of the total chlorophyll content (*Chl* (mg m^−2^), chlorophyll a + chlorophyll b) followed the description in Zhang et al. (2017). All the leaves, including those measured for leaf chlorophyll and *SLA*, on each shoot were then collected and dried in the oven at 65°C for 24 h and weighed for leaf dry mass (*M*_L_, g). The multiples of *M*_L_ and the *SLA* for each shoot were used obtain the total leaf area (*A*_L_, m^2^), which was used to calculate the ratio of leaf area to sapwood area (*A*_L_/*A*_S_, m^2^ cm^−2^).

The dried leaves were ground into powder and analyzed for N content as a mixed sample by an elemental analyzer (EA Flash 1112; Thermo Fisher Scientific). The nitrogen content per unit area (*N* *_a_*, g m^−2^) and the nitrogen content per unit mass were calculated (*N* *_m_*, mg g^−1^).

### The Whole Tree Biomass

To estimate the whole tree biomass, 15 sample trees were selected from each group and harvested to obtained the above-ground and underground total biomass (except fine roots) by calculating the total biomass of the leaves and the root-shoot ratio of each sample tree. Then, we calculated the leaf mass fraction (*LMF*, %) and the leaf area ratio (*LAR*, m^2^ g^−1^), of which total the leaf area is the product of *SLA* and the total biomass.

### Data Analysis and Processing

Analysis of variance (ANOVA) was conducted on all the traits measured above with SPSS 25 (version 25.0, IBM SPSS Inc., Chicago, USA) to examine the treatment effects (*n* = 3) for different species. The relationship between the *SLA*, *Chl*, *A*_max_, and *g*_s_ values and *N* *_a_* and *N* *_m_* across the two species and three treatments were also fitted to analyze the convergence of leaf functional traits due to the elevated N availability. We also regressed *A*_max_ and *K*_S_ with *g*_s_ for the different species in each treatment to verify the variation of the coordination relationship between shoots and leaves in response to N addition. In these analyses, individual sample points (or leaves) were used. Where a significant relation between any two variables was found (*p* < 0.05), we re-analyzed the data with analysis of covariance (ANCOVA) allowing the slope and intercept to vary among treatments. Therefore, in our analysis, a single curve indicates that the treatments or tree species have the same fitted relationship (i.e., the interaction items in the ANCOVA analysis are not statistically significant, *p* > 0.05).

## Results

### Effects of N Additions on Leaf Economics

**Figure 2:**
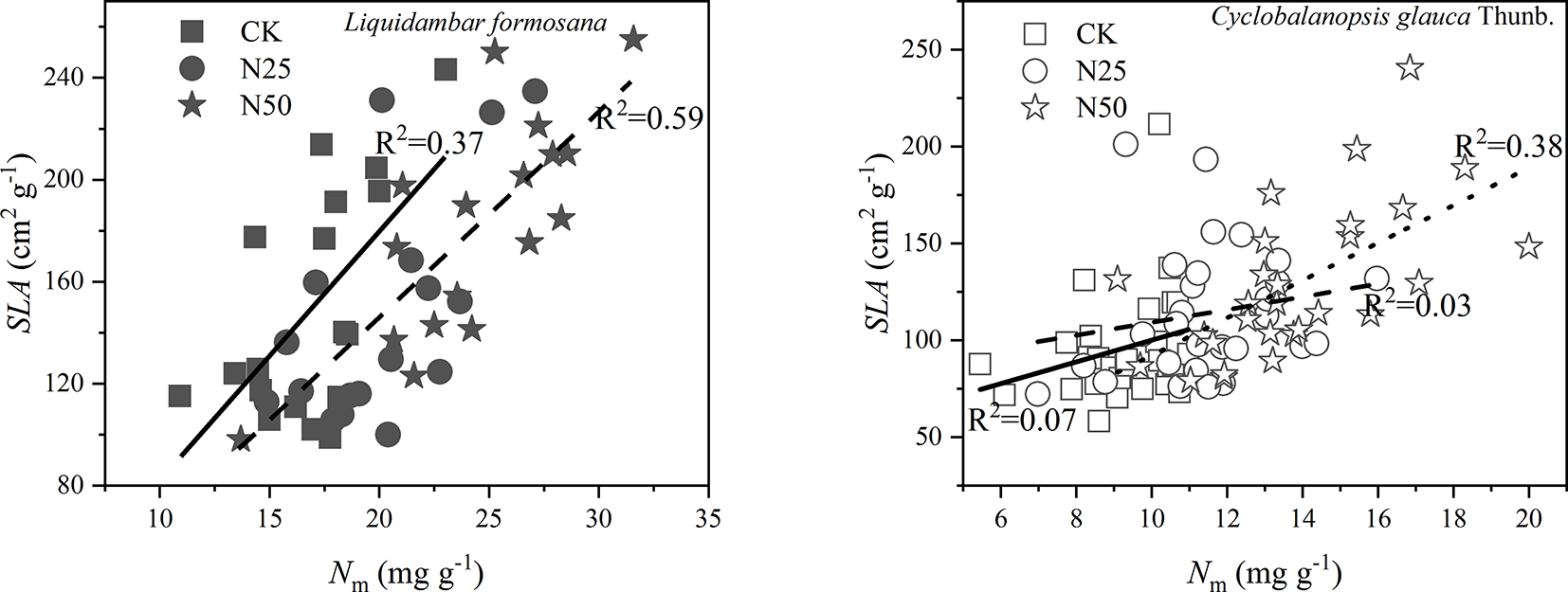
The specific leaf area (*SLA*) as samples of ten leaves averaged for each individual of *Cyclobalanopsis glauca* Thunb. and *Liquidambar formosana* selected for gas exchange measurements as a function of leaf N per unit mass (*N*_m_). Solid lines fit linearly to the CK data, broken lines fit linearly to the N25 data, and dotted line fit linearly to the N50 data. Of note, the data of N25 and N50 share the same relations for *L. formosana*.

As shown in **Figure 2**, the *SLA* variation was well explained by the *N*_m_ of each species along the treatments (p < 0.05 for all the relations based on the ANCOVA analysis). Of note, the data in N25 and N50 shared the same relations for *L. formosana*. The mean *SLA* for *L. formosana* in the highest N treatments (N50) was 184.66 cm^2^ g^−1^, which was 35.99% higher than the unfertilized treatment CK (*p* < 0.01, **Table 1**). Similarly, the mean *SLA* for *C. glauca* increased 34.72% in the N50 treatments compared with the CK treatments (p < 0.01, **Table 1**). In addition, the ratio of leaf area to sapwood area (*A*_L_/*A*_S_) was also enhanced by 26.06% for *L. formosana* but not for *C. glauca* (**Table 1**).

**Table 1:** Treatment means of light-saturated CO_2_ exchange rate (*A*_max_), stomatal conductance (*g*_s_), intercellular CO_2_ (*C*_i_), apparent quantum yield (*AQY*), leaf N per unit leaf mass (*N*_m_) and per unit leaf area (*N*_a_), total amount of chlorophyll (*Chl*) on a mass and area basis, leaf area per mass (*SLA*), hydraulic conductance (*K*_S_), wood density (ρ), and the ratio of leaf area to sapwood area (A_L_/A_S_).

Traits	N treatments	*L. formosana*	*C. glauca*
*A*_max_ (μmol CO_2 m_^−2^s^−1^)	CK	12.63 (2.53)b	7.56 (1.35)a
	N25	11.87 (1.82)b	7.24 (2.16)a
	N50	14.17 (4.03)a	7.91 (2.49)a
*g*_s_ (mol H_2_O m^−2^ s^−1^)	CK	0.12 (0.03)a	0.08 (0.01)a
	N25	0.11 (0.03)a	0.08 (0.02)a
	N50	0.11 (0.01)a	0.05 (0.02)b
*C*_i_ (μmol CO_2_ mol^−1^)	CK	312.55 (15.67)a	327.69 (12.44)a
	N25	307.94 (17.76)ab	211.57 (16.49)b
	N50	276.17 (10.23)b	189.92 (13.55)b
*AQY* (μmol CO_2_ μmol^−1^ PPFD)	CK	0.033 (0.004)b	0.041 (0.013)c
	N25	0.036 (0.011)ab	0.051 (0.017)b
	N50	0.039 (0.014)a	0.059 (0.019)a
*N*_m_ (mg g^−1^)	CK	16.92 (2.82)c	9.24 (0.69)b
	N25	20.09 (3.36)b	11.47 (0.95)ab
	N50	24.37 (4.19)a	13.81 (1.25)a
*N*_a_ (g m^−2^)	CK	1.22 (0.11)a	0.81 (0.32)b
	N25	1.30 (0.25)a	0.95 (0.24)ab
	N50	1.31 (0.22)a	1.07 (0.24)a
*Chl* (mg m^−2^)	CK	2.55 (0.53)b	1.43 (0.36)b
	N25	3.22 (0.78)b	1.62 (0.48)b
	N50	4.39 (0.67)a	2.34 (0.56)a
*SLA* (cm^2^ g^−1^)	CK	135.78 (20.00)b	95.86 (14.73)b
	N25	152.68 (23.34)b	114.14 (16.92)ab
	N50	184.66 (14.67)a	129.14 (19.69)a
*K*_S_ (g cm MPa^−1^ min^−1^ cm^−2^)	CK	4.74 (2.11)b	3.42 (1.41)b
	N25	5.60 (2.24)ab	3.64 (0.86)ab
	N50	7.56 (1.99)a	4.76 (0.67)a
*ρ* (g ml^−1^)	CK	0.53 (0.07)a	0.61 (0.06)a
	N25	0.47 (0.07)b	0.55 (0.06)ab
	N50	0.41 (0.09)b	0.53 (0.04)b
*A*_L_/*A*_S_ (m^2^ cm^−2^)	CK	4.72 (0.79)b	3.56 (0.83)a
	N25	5.18 (1.14)ab	4.24 (0.71)a
	N50	5.95 (0.98)a	3.72 (0.66)a

Different letters indicate differences at p < 0.05, N25, plots with N additions of 25 kg ha^−1^ year^−1^; N50, plots with N additions of 50 kg ha^−1^ year^−1^; CK, control treatments.

The fertilization significantly increased the mean leaf *N*_m_ (*p* < 0.01, **Table 1**) and *N*_a_ (*p* < 0.05, **Table 1**, except for *L. formosana*), which further enhanced the *Chl* in the N50 treatments by 72.15 and 63.63% for *L. formosana* and *C. glauca* respectively. The mean *N*_m_ of *L. formosana* leaves increased 44.03% from 16.92 mg g^−1^ in the CK treatment to 24.37 mg g^−1^ in the highest fertilized N50 treatments. The corresponding increase in *C. glauca* was 49.46% (from 9.24 to 13.81 mg g^−1^). The difference in *N*_a_ between CK and N50 of *L. formosana* was not significant (*p* > 0.05), but it was 32.10% for *C. glauca.*

**Figure 3:**
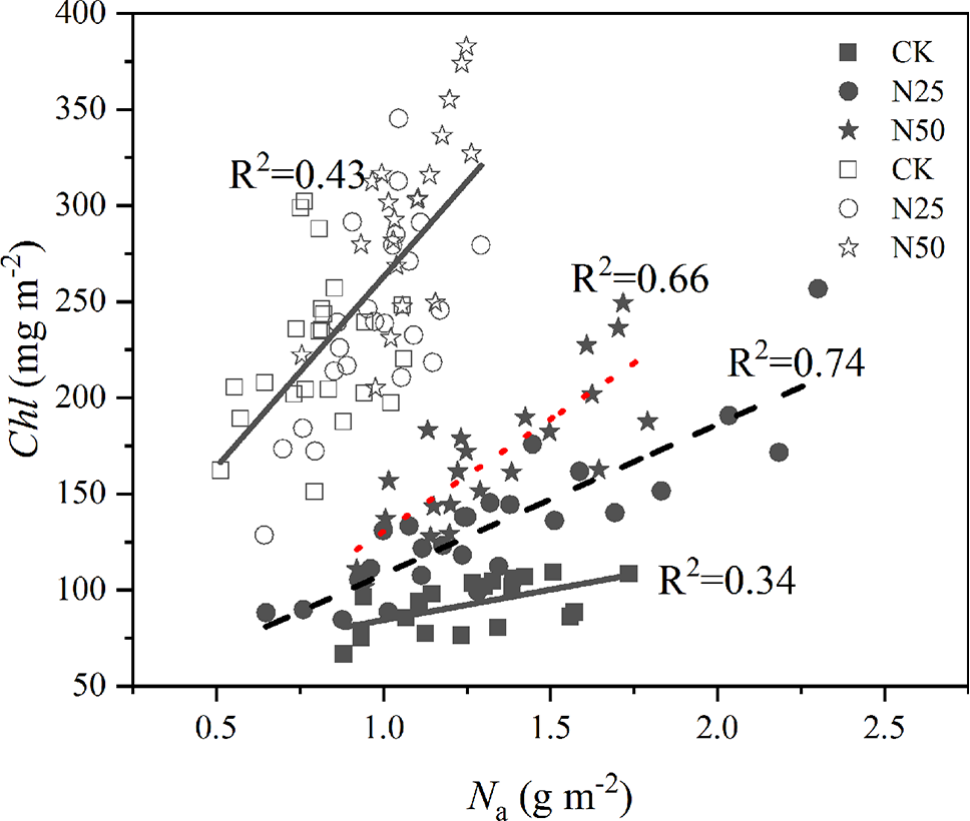
The concentration of *Chl* per unit leaf area as a function of the corresponding measures of leaf N (*N*_a_). Open symbols represent *Cyclobalanopsis glauca*, the closed symbols represent the data of *Liquidambar formosana*. *p* < 0.01 for linear regressions for each species and treatment.

Differences in *N*_a_ across treatments and species were clearly presented by the relations between *Chl* and leaf N (**Figure 3**). For each treatment, the concentration of *Chl* closely responded to the variations of *N*_a_ for the *L. formosana* with different slopes (p < 0.05, ANCOVA), which ranged from the lowest of 31.68 in the CK treatment to the highest of 116.83 in the N50 treatment. The relationship was consistent among treatments for *C. glauca* (*p* < 0.01, ANCOVA). On an area basis, the ratio of *Chl* to *N*_a_ increased 40 and 22.2% for *L. formosana* and *C. glauca* from CK to N50, indicating the higher allocation of newly acquired N to photosynthetic functions for the deciduous *L. formosana*.

### Variation in Photosynthetic Capacity and Water Transport Capacity

**Figure 4:**
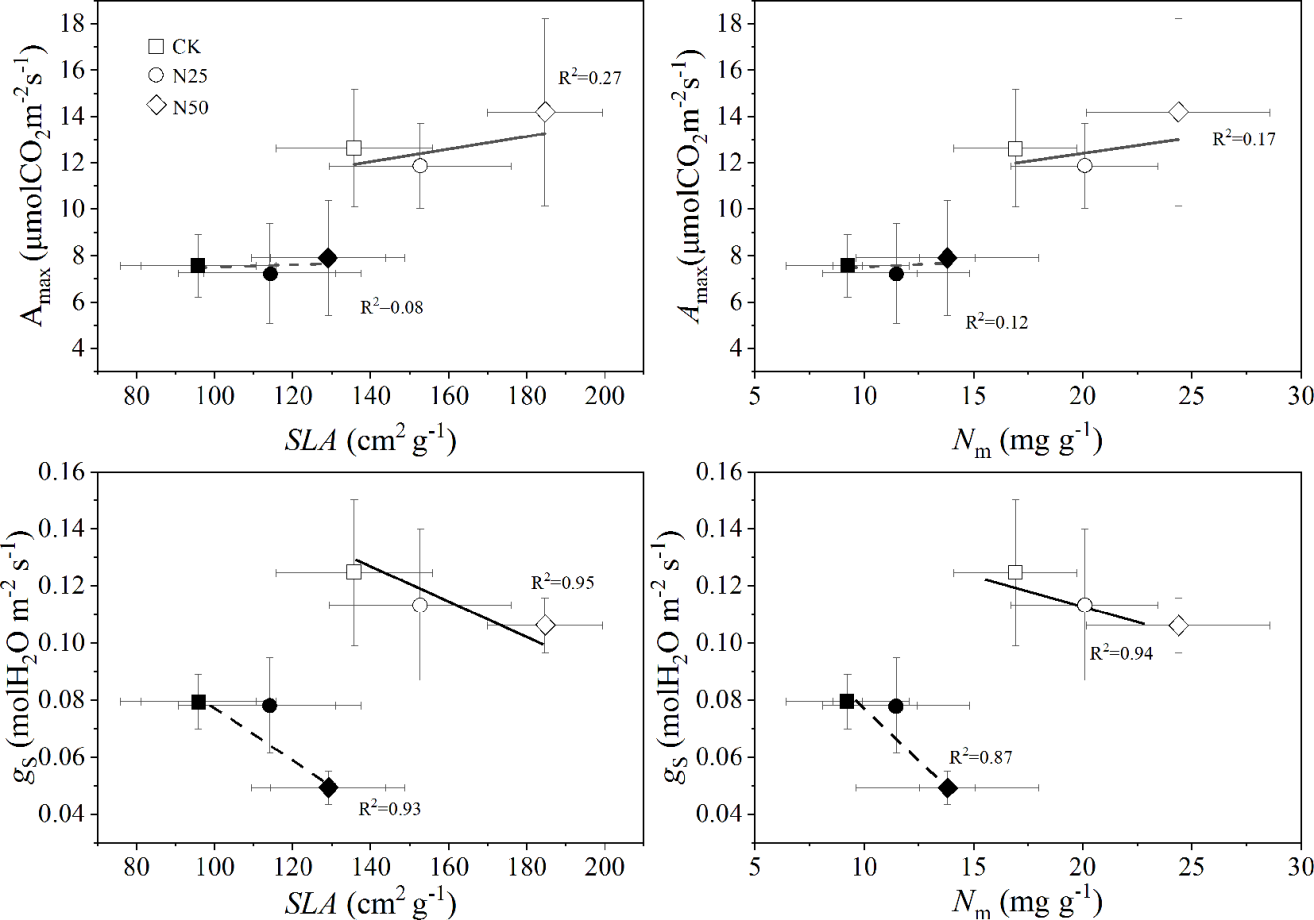
The averaged maximum CO_2_ exchange rate per unit leaf under light-saturated conditions (*A*_max_) and stomatal conductance for H_2_O (*g*_s_) across each light response curve as a function of specific leaf area (*SLA*) and leaf N per mass (*N*_m_). Open and closed symbols represent the data of *Liquidambar formosana* and *Cyclobalanopsis glauca* leaves, respectively. The data are shown as the means ± *SD*.

**Figure 5:**
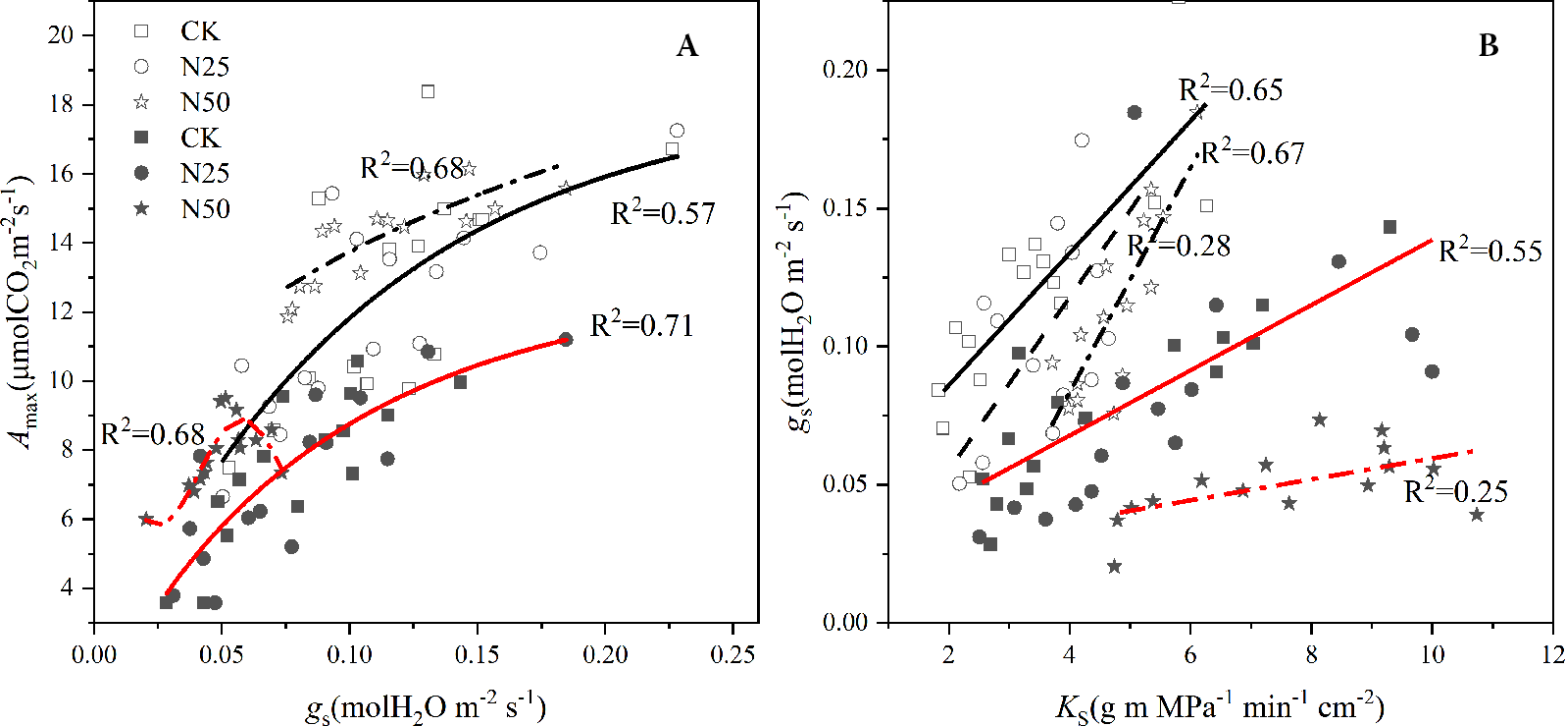
*A*_max_ as a function of mean *g*_s_ for H_2_O averaged over each light response curve and the *K*_S_ averaged from per tree supported *g*_s_ for H_2_O for *Liquidambar formosana* and *Cyclobalanopsis glauca*. Open and closed symbols represent *L. formosana* and *C. glauca*, respectively. p < 0.01 for linear regressions for each species and treatments. Solid lines fit linearly to CK and N25 data, dotted and broken lines fit linearly to N50 data, and the broken lines fit linearly to N25 data for *C. glauca* in the right Figure. Panel **A**: Amax as a function of mean gs, Panel **B**: gs as a function of mean KS.

According to the light response curve, *C*_i_ decreased by 11.64 and 42.04% for *L. formosana* and *C. glauca*, respectively. Meanwhile, *AQY* increased by 15.38 and 43.90%, respectively. The *g*_s_ decreased by 37.5% in the N50 treatments for *C. glauca* but was not significant for *L. formosana* (only 8.33%, *p* > 0.05) (**Table 1**). Among the treatments, the *A*_max_ for *L. formosana* increased 12.19% from CK to N50, whereas this enhancement was not observed in *C. glauca*. Qualitatively, the treatment means of *A*_max_ were found to increase with *SLA* and *N*_m_ for *L. formosana* but were not significant for *C. glauca*, while the *g*_s_ was reduced by the enhancement of *SLA* and *N*_m_ only for *C. glauca* (*p* < 0.05, **Figure 4**). In addition, the relationship between *A*_max_ and *g*_s_ was altered in the N50 (**Figure 5A**), where the *A*_max_ for *L. formosana* tended to be more sensitive to the increase in *g*_s_ when *g*_s_ < 0.15 mol m^−2^ s^−1^, and *A*_max_ for *C. glauca* tended to rapidly decrease when *g*_s_ > 0.06 mol m^−2^ s^−1^ (**Figure 5A**).

In agreement with the enhanced *A*_max_ (albeit not significantly so for *C. glauca*), the water transport capacity per sapwood area (*K*_S_) increased by 59.49 and 39.18% for *L. formosana* and *C. glauca*, respectively. Surprisingly, the improved water transport capacity in the N50 treatments tended to maintain higher and lower *g*_s_ sensitivity for *L. formosana* and *C. glauca*, respectively (**Figure 5B**).

### Daily Dynamics of Gas Exchange and Water Relations

**Figure 6:**
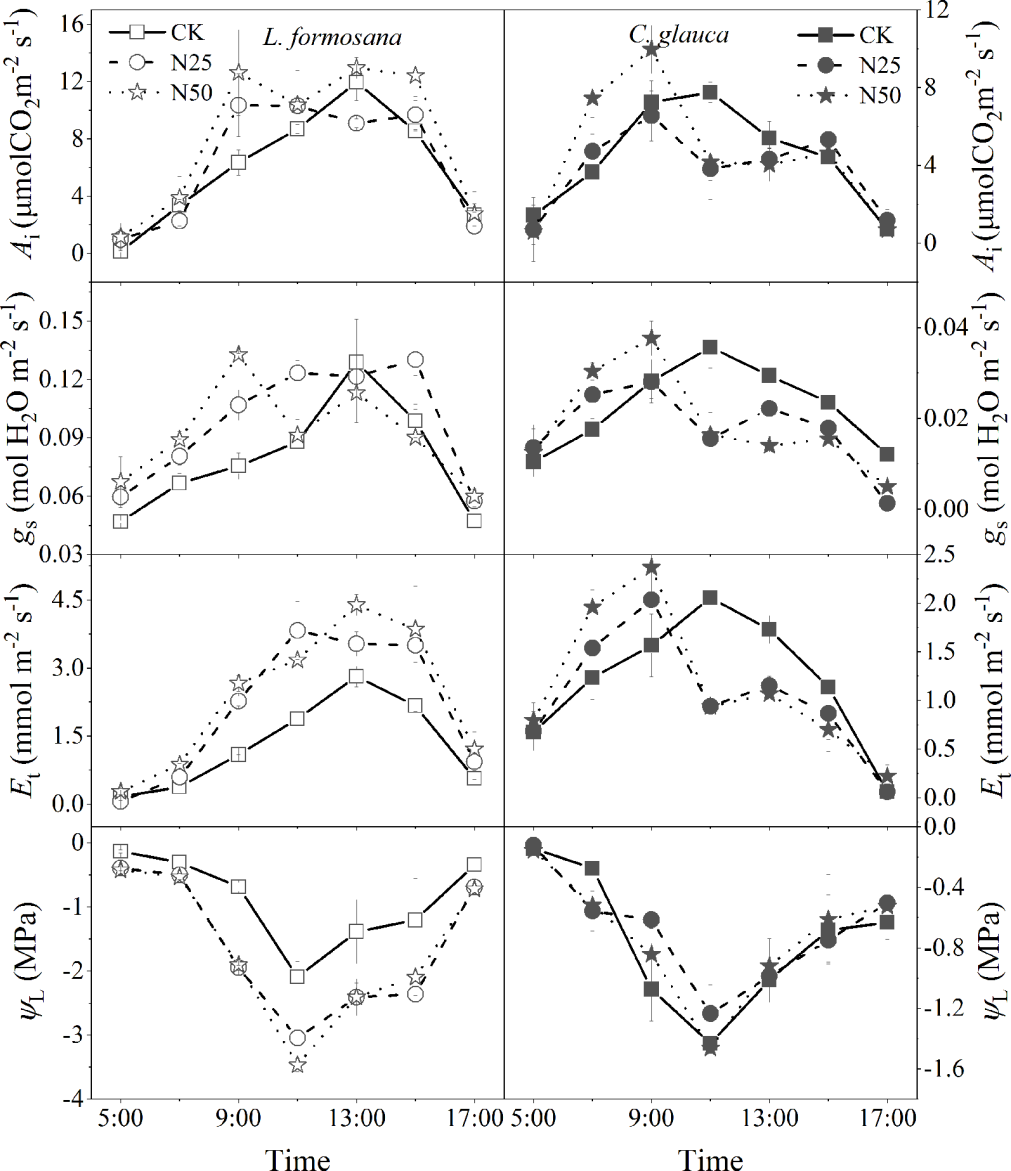
Daily dynamics of leaf instantaneous net photosynthetic rates (*A*_i_), stomatal conductance (*g*_s_), transpiration rates (*E*_t_), and water potential (*Ψ*_L_) across the three N treatments for *Liquidambar formosana* and *Cyclobalanopsis glauca,* respectively. Error bars correspond to the ± standard error of three individuals in each group.

The light was increased on a daily basis before 9:00, and *g*_s_ and *E*_t_ were enhanced in the highest N treatments for both species (**Figure 6**). Notably, even *g*_s_ for both species also experienced a rapid reduction at 11:00, which may be attributed to the lowest *Ψ*_L_ at this time, while the recovery of the *g*_s_ after the replenishment of the *Ψ*_L_ at 13:00 only occurred for *L. formosana*, which also maintained higher *E*_T_ at the same time (**Figure 6**). In contrast, the *g*_s_ and *E*_t_ for *C. glauca* persistently decreased until the end of the experiments. As a result, the *E*_t_ of *L. formosana* in the N50 treatments was apparently enhanced compared to that in the CK treatment, thus leading to excessive water loss (decreased lowest *Ψ*_L_). The reduced *E*_t_ in the N50 from 11:00 to 17:00 for *L. formosana* maintained the water status compared to the CK treatments (stable lowest *Ψ*_L_). These differences enable *L. formosana* to support higher photosynthesis in the N50 treatments, at the cost of exacerbated water stress. In contrast, the *A*_i_ that was elevated by the increased *g*_s_ before 9:00 for *C. glauca* tended to be neutralized after 11:00 in the N50, with the benefits of stable water status (**Figure 6**).

### Variation in Plant Biomass

**Figure 7:**
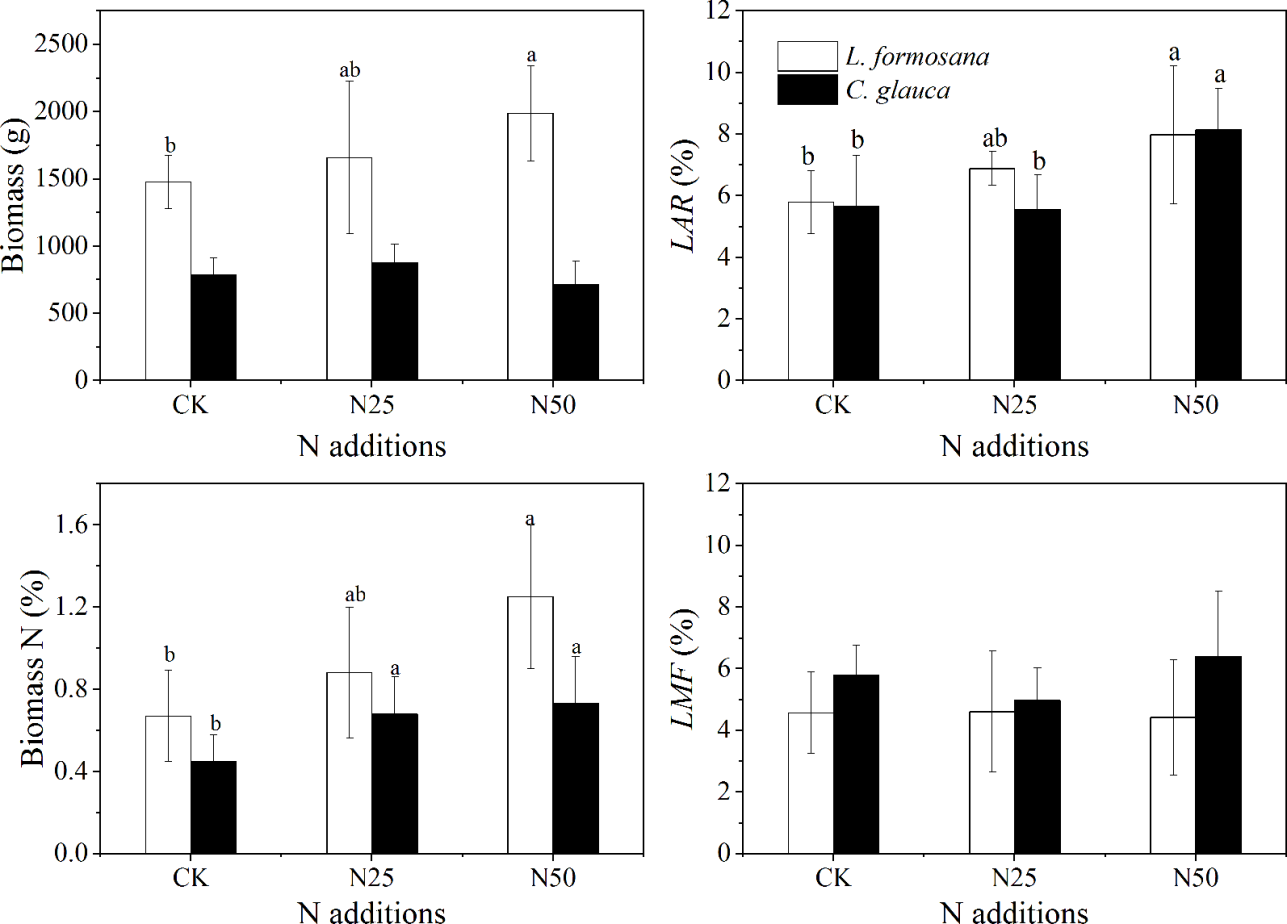
Plant biomass, the fractions of N on the biomass, leaf area divided by total aboveground biomass (*LAR*), and the fraction of leaf mass divided by the total aboveground biomass (*LMF*) of *Liquidambar formosana* and *Cyclobalanopsis glauca* under different N addition treatments: CK, N25, and N50. Plants were harvested from 15 individuals in each of the control and fertilized plots. Error bars represent 1 SD (*n* = 15). Different letters indicate significant differences at *p* < 0.05.

In agreement with the enhanced leaf *N*_m_, the total biomass of *L. formosana* individuals increased by 34.34% in the N50 treatment (**Figure 7**). In contrast, the growth of *C. glauca* was not affected by the enhanced N availability. In addition, the N concentration of plant biomass was found to increase by 86.57% (*L. formosana*) and 62.22% (*C. glauca*) in the N50 treatments. Similarly, the highest N addition rate increased the fraction of leaves to LAR (leaf area divided by total aboveground biomass) by 37.82% for *L. formosana* and 44.15% for *C. glauca*. Notably, the LMF (leaf mass/total aboveground biomass) did not change for either species, which was consistent with the increased leaf SLA across the two species (**Table 1**).

### Hydraulic Changes

**Figure 8:**
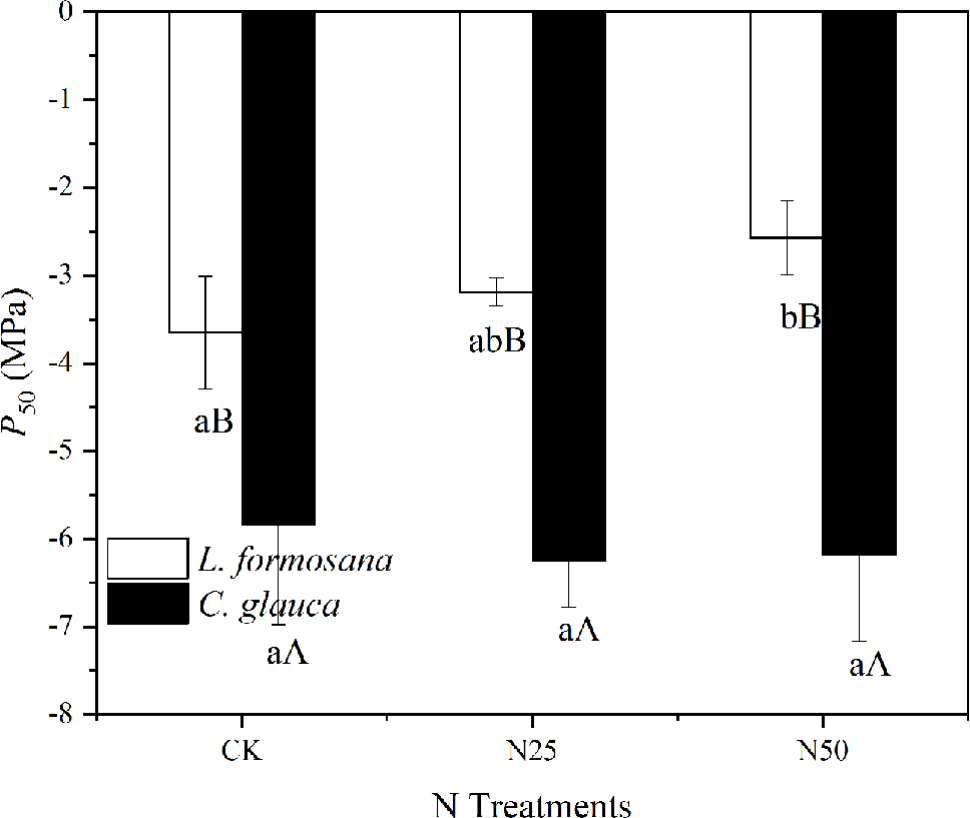
Changes in xylem vulnerability to cavitation *P*_50_ across the three treatments for *Liquidambar formosana* and *Cyclobalanopsis glauca*. Differences between species within treatments and within treatments between species are indicated by different letters (*p* < 0.05).

The *ρ* decreased by 21.56% (*L. formosana*) and 12.95% (*C. glauca*) between the CK treatments and the N50, indicating the reduced mechanical strength for both species due to the enhanced N availability. When the two species were compared, we found that the *P*_50_ was much lower for *C. glauca*, which indicated higher resistance to cavitation (**Figure 8**). *L. formosana* displayed an enhanced xylem vulnerability to cavitation (increased *P*_50_) in the highest N treatments, which is in contrast to *C. glauca*, with weak variations.

## Discussion

### The Accumulation and Allocation of Extra N

In N-saturated ecosystems, the extra N is considered to be weakly accumulated in plant tissues (Curtis and Ackerly, 2008). The subtropical region in China was thought to be N saturated during the past decades (Lu et al., 2014). However, unexpectedly, the mean leaf *N*_m_ and *N*_a_ were obviously enhanced in the N50 treatments for both *L. formosana* (44.03% for *N*_m_) and *C. glauca* (49.46 and 32.10%) (**Table 1**). Meanwhile, the leaf chlorophyll content was improved synchronously and was linearly related to the leaf N across the treatment and species (**Figure 3**), which revealed an enhanced ability to receive light energy, as presented in other studies (Xia and Wan, 2008; Tilman and Isbell, 2015; Wooliver et al., 2016). These results may indicate that the N in the ecosystem was not saturated in this ecosystem, which is contradictory to previous studies conducted in tropical forests (Brookshire et al., 2012; Lu et al., 2018).

In addition, the weak changes in *N*_a_ for *L. formosana* compared to evergreen *C. glauca* contradict our hypothesis, even though the former still has higher leaf N (*p* < 0.01). The reason may be attributed to the increase in *A*_L_:*A*_S_ of 26.06% for *L. formosana*, but not for *C. glauca*, and *N*_m_ increased significantly (**Table 1**). The *A*_L_:*A*_S_ that reciprocal to the Huber value implied the supported leaf area by the sap wood per unit area. This implied the tendency of *L. formosana* to enhance its light acquisition ability to optimize its carbon assimilation (Iversen and Norby, 2008). A study indicated a higher allocation to the photosynthetic apparatus in deciduous species (Takashima et al., 2004), which leads to a weak increase in N allocation per leaf area (**Table 1**).

On the basis of area, the allocation of N to *Chl* was found to vary across the two species (**Figure 3**). The *N*_a_ determined *Chl* was weakly related to the N treatments for *C. glauca*. Similar results have already been reported in another evergreen species, *Eucalyptus cladocalyx* (Myrtaceae) (Simon et al., 2010). *A*_max_ enhanced fraction of *Chl* in the N in the highest N treatments was found for *L. formosana* (**Figure 3**), which is consistent with our hypothesis (Xia and Wan, 2008). The preferential allocation by *L. formosana* leaves of N to chlorophyll synthesis with increasing N fertilization is quite common in deciduous species (Baltzer and Thomas, 2005; Gradowski and Thomas, 2008; Yin et al., 2009). In fact, this evolutionary shift could also be observed in annual herbaceous plants with a similar resource utilization strategy (Feng et al., 2009; Wang et al., 2012a; Mu et al., 2016) but never in evergreen species (Warren and Adams, 2004), which need to invest the N in mechanisms to conserve water and nutrients and to tolerate water and nutrient stress (Warren et al., 2003).

### The Effect of N on Photosynthesis

However, even though both species accumulated N in the leaf biomass and *Chl*, the elevated N was not translated to photosynthesis for *C. glauca* (**Table 1**, **Figure 4**). The *A* *_max_* for *L. formosana* was positively promoted by the enhanced *N*_m_ and *SLA* (*p* < 0.01, R^2^ = 0.27 and 0.17), which is consistent with the resource-limited photosynthesis for most deciduous species in previous studies (Xia and Wan, 2008; Kröber and Bruelheide, 2014; Wooliver et al., 2016). In contrast, the *A*_max_ for the evergreen *C. glauca* did not respond to N accumulation. The significantly increased *AQY* indicated the enhanced capacity to assimilate CO_2_ for both species, which was consistent with the accumulated *Chl* in the N50 treatment across the two species (**Table 1**, **Figure 3**). However, the large proportion of decreased *C*_i_ for *C. glauca* (by 42.04%) may imply severe stomatal limitation when the *L. formosana* was compared (11.64%), thus eliminating the N effect on photosynthesis. Indeed, studies have indicated that the effect of nitrogen on photosynthesis of evergreen species will be enhanced by elevated CO_2_ (Bauer et al., 2001). Thus, we can expect that the stomatal-limited CO_2_ could explain the weak changes of *A*_max_ for the evergreen *C. glauca*.

### Hydraulically Limited Plant Biomass Accumulation

The stomatal limitations did occur for *C. glauca* in the highest N treatments. The *g*_s_ in the N50 treatments decreased by 37.5% for *C. glauca* and tended to be less related to the sapwood conductivity (**Figure 5**). In contrast, the *g*_s_ tended to be more and more sensitive to the increase of *K*_S,_ along with the weak decrease (by 8.33%, *p* > 0.05) for *L. formosana*. The *K*_S_ for both species increased significantly (by 59.49 and 39.18%). We expected that the reduced *g*_s_ may be attributed to the enhanced water lose on the leaf supported by the increased *K*_S_ and *SLA*. In dry conditions, the increased *AQY* will raise photosynthesis and leaf transpiration under low light conditions, which further lead to excessive water loss and severe stomatal control for the evergreen *C. glauca* (**Figure 6**). In addition, the increased *SLA* also added to the water loss, which elevated the light acquisition ability as well as the transpiration area. However, stomatal limitation did not occur for *L. formosana*, and a similar increase in *SLA*, *AQY*, and *K*_S_ was observed (**Table 1**, **Figure 6**), which was thought to be related to the different water use regulation strategies for the evergreen and deciduous species. Evergreen species tended to rapidly close their stomata in response to excessive water loss to maintain higher leaf water potential in contrast to the deciduous species (**Figure 6**), which is known as isohydric-prone *versus* anisohydric-prone behavior (Kumagai and Porporato, 2012; Klein and Niu, 2014; Siddiq et al., 2017). The first will reduce the risk of damaging xylem cavitation driven by excessive tension in the trees’ hydraulic system (Klein and Niu, 2014). However, a consequence of this strategy is that these trees close their stomata in response to even mild water stress—a process that reduces leaf carbon (C) uptake (Siddiq et al., 2017). In our study, the leaf scale photosynthesis was translated into the accumulation of whole tree biomass (by 34.34%) for *L. formosana* in the N50 treatments but not for *C. glauca*, even though the N concentration of plant biomass for both species was apparently elevated. These results indicated that the prevailing seasonal drought did not neutralize the fertilization effect for *L. formosana* but had the inverse effect in *C. glauca*.

It is noted that the increase in biomass observed for *L. formosana* does not imply certain success in species competition. In fact, even though anisohydric trees could allow their leaf Ψ (*Ψ*_L_) to decrease during drought by sustaining relatively high g_s_ (and thus C assimilation), a greater risk of cavitation in the xylem could ultimately lead to rapid declines in leaf water supply that may affect a range of physiological variables, including photosynthetic capacity and g_s_ (McDowell et al., 2008; Roman et al., 2015). In fact, the *P*_50_ of *C. glauca* almost doubled that of *L. formosana*, thus revealing advantages in response to seasonal drought. Furthermore, the elevated N availability tended to enhance the leaf water loss by increasing LAR and weakly changing LMF for both species, while the *P*_50_ was elevated only for *L. formosana*. Thus, extreme drought events may threaten the survival of *L. formosana via* hydraulic failure (Roman et al., 2015), especially in the context of elevated N availability.

## Conclusion

In our study, the two species that belong to two different forest types behave distinctly in response to elevated N availability. Both the deciduous *L. formosana* and the evergreen *C. glauca* accumulated N in *Chl*, which led to elevated *AQY* in the leaves. In combination with the increased *SLA* and whole tree leaf area, the transpiration demands were promoted for both species. However, the lower compensation of elevated *K*_S_ could not balance the excessive leaf water loss, which lead to decreased g_s_ for the evergreen *C. glauca*. In contrast, the elevated A_n_ for the deciduous *L. formosana* accumulated a 34.34% increase in whole tree biomass. However, due to the anisohydric behavior and less negative *P*_50_, especially when N was elevated for *L. formosana*, the competitive relationship between the two species is still inconclusive due to the risk of hydraulic failure in the face of the gradually enhanced seasonal drought in the Yangtze River Delta.

## Data Availability Statement

All datasets generated for this study are included in the article/supplementary material.

## Author Contributions

ZZ designed the experiments. YZ, XZ, and ST conducted the experiment, XF, YC, LZ, XL and CW conducted the statistical analyses of data. ZZ and YZ wrote the manuscript. All authors read and approved the final manuscript.

## Funding

The study was supported by the National Nature Science Foundation of China (Grant No. 41701226) and the Zhejiang Province Public Welfare Technology Application Research Project (LGF19C030002). Jinhua Science and Technology Research Project (2019-4-163). Data used in this study were collected by the author and are available from the author upon request.

## Conflict of Interest

The authors declare that the research was conducted in the absence of any commercial or financial relationships that could be construed as a potential conflict of interest.
